# Combined suppression effects on hydrodynamic cavitation performance in Venturi-type reactor for process intensification

**DOI:** 10.1016/j.ultsonch.2022.106035

**Published:** 2022-05-13

**Authors:** Mingming Ge, Chuanyu Sun, Guangjian Zhang, Olivier Coutier-Delgosha, Dixia Fan

**Affiliations:** a Research Center of Fluid Machinery Engineering and Technology, Jiangsu University, Zhenjiang 212013, China; b School of Engineering, Westlake University, Xihu District, 310024 Hangzhou, China; c Kevin T. Crofton Department of Aerospace and Ocean Engineering, Virginia Tech, Blacksburg, VA 24060, USA; d Section of “Chemistry for the Technology” ChemTech, Department of Industrial Engineering, University of Padova, I-35131 Padova (PD), Italy

**Keywords:** Hydrodynamic cavitation, Venturi reactor, Cavitation intensity, Spatio-temporal distribution, Thermodynamic effects, Process intensification

## Abstract

Hydrodynamic cavitation is an emerging intensification technology in water treatment or chemical processing, and Venturi-type cavitation reactors exhibit advantages for industrial-scale production. The effects of temperature on hydrodynamic cavitating flows are investigated to find the optimum reaction conditions enhancing cavitating treatment intensity. Results show that the cavitation performance, including the cavitation intensity and cavitation unsteady behavior, is influenced by (1) cavitation number σ (the pressure difference affecting the vaporization process), (2) Reynolds number *Re* (the inertial/viscous ratio affecting the bubble size and liquid–vapor interface area), and (3) thermodynamic parameter Σ (the thermal effect affecting the temperature drop). With increasing temperature, the cavitation length first increases and then decreases, with a cavitation intensity peak at the transition temperature of 58 °C. With the growth of cavitation extent, the cavity-shedding regimes tend to transition from the attached sheet cavity to the periodic cloud cavity, and the vapor volume fluctuating frequency decreases accordingly. A combined suppression parameter (*CSP*) is provided to predict that, with increasing *CSP* value, the cavitation intensity can be decreased. Recommendations are given that working under the low-*CSP* range (55–60 °C) could enhance the intensification of the cavitation process.

## 1 Introduction

Cavitation, as a greener processing technique, has been utilized for a wide variety of extraordinary applications, such as wastewater treatment [1], dye degradation [2], crude oil demulsification [3], and extraction of bioactive compounds from green tea [4], among others. Among these processes, the collapse of bubbles in cavitation creates extreme conditions at localized areas with high-temperature “hotspots” up to 5000 K, high pressures up to 1000 bar, as well as high oxidation (hydroxyl radicals) [5]. This kind of sonochemical mechanism can be used for process intensification to enhance the reaction rates or the yields of production due to its direct (mechanical, thermal, and chemical) and secondary effects, including turbulence, mechanical vibration, macroscopic heating, emulsification, and dispersion [6].

Hydrodynamic cavitation (HC), generated by locally accelerated flow, is quite suitable for large-scale industrial applications [7], [8]. Compared with other generation methods, such as optic, acoustic, and particle, HC offers the advantages of good scalability, inexpensive device fabrication, and promising treatment effect [9]. The efficiency (e.g., oxidation rate and pre-treatment rate) of HC is related to various parameters affecting cavitation, including geometry, flow velocity, pH, temperature, inlet pressure, gas content, reagent concentration, kind and concentration of biomass, and time of reaction; thus, optimization is needed [10]. However, from the viewpoint of cavitation itself, all the aforementioned external factors essentially influence HC performance by changing the cavitation intensity and its dynamical behavior.

The term “cavitation intensity” has been used to characterize the aggressiveness or strength of cavitation for decades [11]. Most of the time, cavitation intensity is used as self-evident terminology and previous definitions somehow seem either biased or vague. Indeed, no unanimous consensus has been reached for the definition of cavitation intensity [12]. To measure or calculate the cavitation intensity, various methods have been developed based on different experimental conditions. For instance, Raviyan et al. [13] estimated cavitation intensity by measuring hydrogen peroxide ($H_{2}O_{2}$) formation in distilled water during sonication. In [14], Choi et al. conducted systematic erosion tests to correlate materials’ erosion levels with empirically accepted cavitation intensity indicators, such as flow speed, ambient pressure, amplitude, and frequency of an ultrasonic horn. Considering the energy, Wu et al. [12], [15] defined cavitation intensity using the cavitation energy $E_{\mathrm{ca}}$ unit time and unit space, which represents the power density of cavitation. However, the above-mentioned methods are either too specific to be compared with each other or impractical to use in the engineering field. To unify these definitions of “cavitation intensity” is difficult and out of the scope of this paper, but we choose to relate the cavitation intensity with the extent (size, length, thickness, area, or volume in three dimensions) of cavitation [16], [17], [18]. It is widely observed from the above literature that a larger extent of cavitation leads to stronger influences on water treatments and erosion rates. However, this correlation is natural and simple.

Researchers [19], [20] found that the operating conditions, e.g. Reynolds number *Re* and cavitation number σ, affect cavitation intensity and its dynamical behavior. However, the majority of studies on cavitation control have assumed isothermal conditions without considering thermal effects. At high temperatures, specific thermodynamic properties, such as the steep slope of saturation pressure–temperature curves, low thermal conductivity, and low liquid–vapor density ratios, cannot be negligible, contrary to cold water. In hot water, the larger mass transfer and subsequent sensible-latent heat conversion rates are needed to sustain the cavity vapor with comparable sizes. The latent heat must be absorbed from surrounding liquids during the vaporization process of bubble growth. Consequently, substantial evaporative cooling effects occur surrounding the cavity in water, and a few degrees temperature difference can be expected between the liquid bulk and cavitating region. Thus, a delay of cavitation can be expected due to the temperature difference between the mainstream liquid and local cavity area, i.e., the thermodynamic effect [21].

Experiments were performed for diverse liquids from refrigerants to water on diverse geometries to investigate their thermodynamic effects [22]. Although widely observed for thermal delay phenomena, the consensus has not been reached for the effects of temperature on cavitation characteristics such as size, erosion rate, regimes, and dynamics behaviors. With measurements in various flow conditions and temperature ranges, some researchers reported that the cavitation length decreased [23], [24] or increased [25] monotonously with increasing temperature, while others observed that it is a “first increase, then decrease” trend [26], [27], and the thermal effects become significant at a transition temperature. The maximum cavitation intensity was reported in a large range from 40 °C to 70 °C, even for the pure water that has mostly been investigated [28], [29]. Moreover, the temperature effects on cavitation dynamics such as vapor cloud shedding and collapsing behavior are still not elucidated.

A concise review of previous works shows that cavitation has advantages in many processing applications such as emulsification, biodiesel preparation, wastewater decontamination, and organic synthesis as a green intensification method [30]. However, investigations on effective factors (e.g. temperature effect) to control and the mechanisms to predict the cavitation intensity, especially in the Venturi device, still remain rare. For this reason, a convergent/divergent Venturi nozzle is used to generate hydrodynamic cavitation in different shedding regimes. To investigate the effects of temperature on hydrodynamic cavitation, a heating and cooling functioned loop was used to change the water temperature between 28 °C and 72 °C. Together with adjusting the flow velocity and the freestream pressure as explained in Section 3, the flow conditions, e.g., cavitation number, Reynolds number, and thermal parameters, are under control. Section 2 is focused on the quantifying methods of the thermal effect of cavitation in history and provides a new combined suppression parameter (*CSP*). Section 4 presents the suppression effects on cavity growth and highlights the effectiveness and practicability of using *CSP* for cavitation intensity quantification.

## 2 Theoretical background

Because of the differences between the bulk flow temperature and local cavity temperature, a delay of cavitation intensity (indicated by cavitation number) can be expected. The cavitation number σ characterizes the potentials of a liquid to cavitate, which can be scaled as a non-dimensional ratio between fluid dynamic pressures and pressure differences.

(1) $$\sigma =\frac{P_{\mathrm{ref}}-P_{\mathrm{vap}}\left(T_{\infty }\right)}{\frac{1}{2}\;\rho U_{\mathrm{ref}}^{2}}$$

Here, ρ is the liquid density, $P_{\mathrm{vap}}\left(T_{\infty }\right)$ the vapor pressure at the freestream bulk temperature, $P_{\mathrm{ref}}$ the upstream pressure, and $U_{\mathrm{ref}}$ the flow velocity at the Venturi throat. However, the local temperature drops from the thermal effect indicate that the cavitation initiates at a local vapor pressure. The cavitation number for cavity regions $\sigma_{c}$ can be written as

(2) $$\sigma_{c}=\frac{P_{\mathrm{ref}}-P_{\mathrm{vap}}\left(T_{c}\right)}{\frac{1}{2}\rho U_{\mathrm{ref}}^{2}}$$

where $T_{c}$ is a local temperature at cavity regions. A first-order approximation can be applied to relate these two cavitation numbers as follows:

(3) $$\frac{1}{2}\rho_{l}U_{\mathrm{ref}}^{2}\left(\sigma_{c}-\sigma \right)=\frac{dp_{v}}{\mathrm{dT}}\left(T_{c}-T_{\infty }\right)$$

in which $\frac{dp_{v}}{\mathrm{dT}}$ can be read from the vapor-pressure–temperature curve. The cavitation intensity loss can be reflected by the increase of the effective cavitation number due to the local temperature drop $\Delta T=T_{c}-T_{\infty }$.

Efforts have been made to quantify this temperature drop in cavitation using fluid and vapor properties at a given temperature. In [31], researchers introduced a B factor developed from the process that “the thermal suppression to be cooled for liquid” equals “heat to be supplied for vaporization” (Eq. 4). B is the ratio between the liquid volume lost and the vapor volume produced during the vaporization process (Eq. 5). Then, [32] introduced several semi-empirical values of the B factor from experimental results obtained in different liquids. With a known B factor, the scale of temperature difference ΔT can be used to quantify the thermal effect in the vaporization/cooling process (Eq. 5).

(4) $$\rho_{v}v_{v}L=\rho_{l}v_{l}C_{\mathrm{pl}}\Delta T$$

(5) $$\Delta T=B\times \Delta T^{*}\text{;}\;\mathrm{where},B=\frac{v_{v}}{v_{l}},\Delta T^{*}=\frac{\rho_{v}L}{\rho_{l}C_{\mathrm{pl}}}$$

Here, *L* is the latent heat, $C_{\mathrm{pl}}$ the specific heat of water, $\rho_{v}$ and $\rho_{l}$ are densities, and $v_{v}$ and $v_{l}$ volume flow rates for the vapor phase and the liquid phase, respectively. With the calculation of $\Delta T^{*}$ and multiple ways of estimating *B* based on liquid properties and cavity sizes, the temperature drop ΔT can be estimated.

Other non-dimensional terms are provided to characterize the temperature drop. The Jakob number, which is a reciprocal of the B factor mathematically, compares the ratio between the latent heat needed to generate bubbles through a phase change $\left(V_{b}\rho_{G}L\right)$ and the sensible heat available in the volumes of liquid regarding the bubble volumes $\left(V_{b}\rho_{l}C_{\mathrm{pl}}\left(T_{l}-T^{\mathrm{sat}}\left(p_{L}\right)\right)\right)$. As reported by [33], the B factor is usually employed when investigating cavitation with the thermal effect, while a Jakob number is often used to study phase change in boiled fluids. Additionally, the C factor, was proposed by [34], which can associate the thermodynamic effects with the thermal transition time during cavitation of different thermo-fluids.

Although these parameters can be used to estimate the local temperature drop reasonably, the impacts of temperature on the overall cavitation evolution trend and the cavity dynamic behavior are inadequate to be predicted [35]. No reliable correlation for the cavity growth and temperature is presently available in addition to achieving thermal control of cavitation intensity quantitatively.

**Fig. 1:**
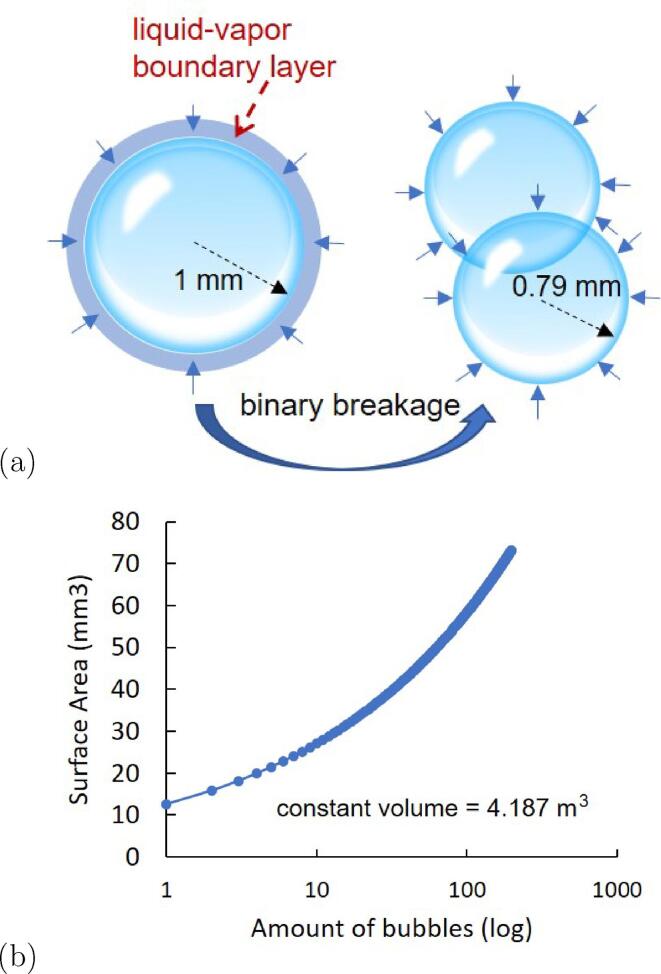
Binary breakage of bubbles with constant vapor volume: (a) Schematic of bubble separation; (b) surface area increasing with number of bubbles for constant vapor volume.

In practice, the development of cavitation is affected by multiple factors, such as pressure difference, turbulence level, and thermal effects. The most significant factor, i.e. the pressure difference between the vapor pressure and local pressure, can be represented by the cavitation number σ, which has been widely investigated [20]; it has also been observed in our study that with smaller σ the cavitation tends to be longer and thicker, and finally cloud cavitation is activated. If we keep the σ number the same in all experimental sets to eliminate the effect of the pressure difference, the temperature effect can be investigated. At different temperatures, two other mechanisms, i.e. the viscous effect and the thermal effect, must be considered. Viscosity decreases with increasing temperature, and thus the Reynolds number (Re) increases. As widely accepted in computational fluid dynamics (CFD) simulation [36] and observed in experiments [19], lower viscosity, and hence higher Re, experiences the so-called turbulence effect, which could enhance cavitation. [37], [38] treated this turbulence effect by increasing the phase-change threshold pressure value as $P_{v}=\left(P_{\mathrm{sat}}+P_{\mathrm{turb}}^{\prime }/2\right)$, where $P_{\mathrm{turb}}^{\prime }=0.39\rho k$, and *k* denotes turbulent kinetic energy. They statistically related the estimation of turbulent pressure fluctuations with computations of time-averaged phase-change rates, based on the probability-density-function (PDF) approach. Based on the statistical models of breakage rates and daughter-size distributions for droplets in a turbulent flow [39], the bubble size tends to be smaller for higher turbulent flows and mainly binary breakage occurs for the bubbles within the fluid. Thus, a larger number of bubbles is generated in a constant volume of vapor. The area of the interface between the liquid and vapor expands following the curve in Fig. 1, which is calculated based on the assumption of a perfectly spherical bubble originally with a 1-mm radius. In the upstream, where evaporation dominates, the larger interface provides a sufficient boundary-layer zone for the liquid evaporating into the vapor, which creates both a longer cavity length and higher intensity.

The turbulence effect explains the increasing trend of cavitation growth and transition. However, the thermal effects must also be considered, as the energy consumed during the vaporization process could delay the growth of cavitation, especially at higher temperatures. Many parameters have been proposed to quantify the thermal effects as mentioned. The thermodynamic parameter Σ (Eq. 6) proposed by [40] and its non-dimensional form Σ* (Eq. 7) introduced by [41] are used here to assess the thermodynamic effect. As suggested by [23], if the non-dimensional quantity Σ* values are consistent, two thermally dominated flows can be dynamically similar.

(6) $$\Sigma =\frac{\rho_{v}^{2}L^{2}}{\rho_{l}^{2}c_{\mathrm{pl}}T_{\infty }\sqrt{\alpha_{l}}}$$

(7) $$\Sigma^{*}=\frac{\Sigma \cdot H_{\mathrm{th}}^{1/2}}{U_{\mathrm{th}}^{3/2}}$$

Here, $\rho_{l}$ is the liquid density, $\alpha_{l}$ the thermal diffusivity of the liquid, $c_{\mathrm{pl}}$ the specific heat of water at constant pressure, $\rho_{v}$ the vapor density, *L* the latent heat of vaporization, and $T_{\infty }$ the liquid temperature. $H_{\mathrm{th}}$ is the throat height of the channel while $U_{\mathrm{th}}$ is the average flow velocity through it. The physics behind this thermodynamic parameter starts with studying a single spherical bubble evolution. Heat transfer is achieved only through the conduction in liquids, and non-slip is assumed between the liquids and the bubbles. Fourier’s law is used to estimate the conductive heat flux per unit surface area at the liquid-bubble interface:

(8) $$q=\frac{\overset{˙}{Q}}{A}=\frac{\Delta Q}{4\pi R^{2}\Delta t}\approx -\lambda_{l}\frac{\Delta T}{\Delta x}$$

in which $\Delta T=T_{c}-T_{\infty }$ represents the temperature differences of the cavitation bubbles and the freestream; $\Delta x\approx \sqrt{\alpha_{l}\Delta t}$ is the characteristic thickness of the thermal boundary layer at the bubble interfaces. To estimate the heat transfer ΔQ, the bubble growth can be treated as a combination of the (1) gas expansion/compression and (2) phase-change processes. First, the gas expansion/compression process considers only non-condensable gas without phase change.

(9) $$\left(\frac{4}{3}\pi R^{3}\right)\rho_{g}c_{\mathrm{vg}}\Delta T=\Delta Q-p\left(4\pi R^{2}\Delta R\right)$$

Here, R is the bubble radius. As investigated by [42], the characteristic time of the heat-transfer process is on the order of 0.5 ns, which is far smaller than the typical lifetime of a bubble in water growing and collapsing around 10–100 ms, depending on the bubble size. Therefore, during such a short heat-transfer period, the bubble-size evolution can be assumed to be mainly governed by a thermal equilibrium of the phase change due to vaporization/condensation:

(10) $$\Delta Q=q\cdot 4\pi R^{2}=\frac{d}{\mathrm{dt}}\left(\frac{4}{3}\pi R^{3}\right)\rho_{v}L$$

(11) $$q=\rho_{l}L\overset{˙}{R}$$

The heat transfer ΔQ and heat flux q can be derived from Eq. 8 as well:

(12) $$\Delta Q\approx -\lambda_{l}\frac{\Delta T}{\sqrt{\alpha_{l}\Delta t}}4\pi R^{2}\Delta t=-\sqrt{\lambda_{l}\rho_{l}c_{\mathrm{pl}}}\sqrt{\Delta t}4\pi R^{2}\Delta T$$

(13) $$q\approx -\lambda_{l}\frac{\Delta T}{\sqrt{\alpha_{l}\Delta t}}\Delta t=-\sqrt{\lambda_{l}\rho_{l}c_{\mathrm{pl}}}\sqrt{\Delta t}\Delta T$$

Combining Eqs. (11), (13), the temperature difference ΔT can be calculated based on bubble-size evolution:

(14) $$\Delta T=T_{b}-T_{\infty }=-\frac{\overset{˙}{R}\sqrt{\Delta t}}{\sqrt{\alpha_{l}}}\frac{\rho_{v}L}{\rho_{l}c_{\mathrm{pl}}}$$

Considering the thermal effect in the Rayleigh-Plesset equation (Eq. 15), the global vapor pressure $p_{v}\left(T_{\infty }\right)$ is changed to local vapor pressure $p_{v}\left(T_{c}\right)$. As stated by [43], after the micro-bubbles becomes macroscopic cavitation, the Rayleigh-Plesset equation can be simplified into the Rayleigh equation (Eq. 16), in which the effects of non-condensable gases, viscosity, and surface tension are not considered.

(15) $$\rho \left[R\overset{¨}{R}+\frac{3}{2}{\overset{˙}{R}}^{2}\right]=\left[p_{v}(T_{\infty })-p_{\infty }\right]+p_{g0}{\left(\frac{R_{0}}{R}\right)}^{3k}-\frac{2S}{R}-4\mu \frac{\overset{˙}{R}}{R}$$

(16) $$\rho \left[R\overset{¨}{R}+\frac{3}{2}{\overset{˙}{R}}^{2}\right]=\left[p_{v}(T_{c})-p_{\infty }\right]$$

Owing to the difficulty of measuring the cavity temperature $T_{c}$, the local vapor pressure $p_{v}(T_{c})$ can be estimated using Eq. 17. Based on the temperature difference ΔT derived in Eq. 14, the Rayleigh equation (Eq. 16) can be rewritten as Eq. 18, in which the cavity temperature is not explicitly used.

(17) $$p_{v}\left(T_{c}\right)=p_{v}\left(T_{\infty }-\Delta T\right)=p_{v}\left(T_{\infty }\right)-\Delta p_{v}\approx p_{v}\left(T_{\infty }\right)-\frac{{\mathrm{dp}}_{v}}{\mathrm{dT}}\Delta T$$

(18) $$\left(R\overset{¨}{R}+\frac{3}{2}{\overset{˙}{R}}^{2}\right)+\frac{{\mathrm{dp}}_{v}}{\mathrm{dT}}\frac{\overset{˙}{R}\sqrt{t}}{\sqrt{\alpha_{l}}}\frac{\rho_{v}L}{\rho_{l}c_{\mathrm{pl}}}\frac{1}{\rho_{l}}=\frac{p_{v}\left(T_{\infty }\right)-p_{\infty }}{\rho_{l}}$$

In Eq. 18, the second term is the thermal effect term by which temperature affects the bubble evolution. Letting $\Sigma =\frac{\rho_{v}L}{\rho_{l}^{2}c_{\mathrm{pl}}\sqrt{a}}\frac{{\mathrm{dp}}_{v}}{\mathrm{dT}}$ and using the Clausius–Clapeyron relation $L=T\left[\frac{1}{\rho_{v}}-\frac{1}{\rho_{l}}\right]\frac{dp_{v}}{dT}\approx \frac{T}{\rho_{v}}\frac{dp_{v}}{dT}$, the final equation, Eq. 19, can be used to describe the cavity dynamics that includes the thermal effects.

(19) $$R\overset{¨}{R}+\frac{3}{2}{\overset{˙}{R}}^{2}+\Sigma \overset{˙}{R}\sqrt{t}=\frac{p_{v}\left(T_{\infty }\right)-p_{\infty }}{\rho_{l}},\;\mathrm{in}\;\mathrm{which},\;\Sigma =\frac{{\left(\rho_{v}L\right)}^{2}}{\rho_{l}^{2}c_{p_{l}}T_{\infty }\sqrt{\alpha_{l}}}$$

**Fig. 2:**
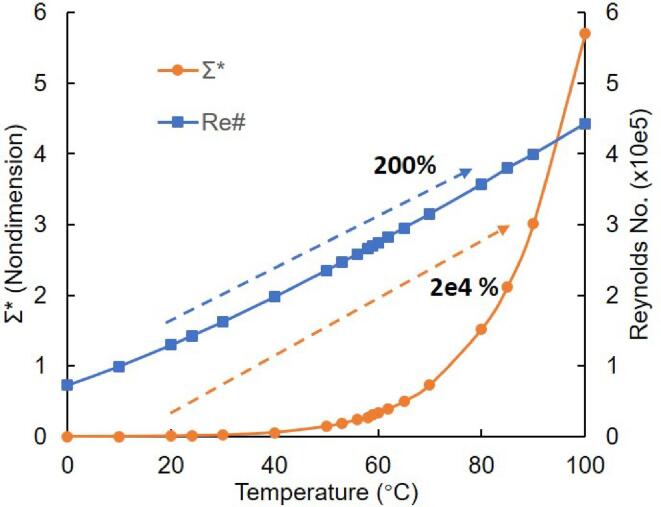
Thermodynamic parameter and Reynolds number variation along with temperature in water.

This thermodynamic parameter Σ consists of property parameters of vapor and liquid, which is a thermo-physical quantity that purely depends on the temperature only. Thus, thermal delay from various liquids can be compared considering that a larger Σ (or $\Sigma^{*}$) denotes a stronger thermodynamic effect [44]. As shown in Fig. 2, the $\Sigma^{*}$ value surges by 200 times compared to twice the *Re* number, when the temperature increases from 20 °C to 80 °C. The *Re* number is scaled by dividing $10^{5}$ to be of the same order as $\Sigma^{*}$. At low temperatures, the variation of the *Re* number mainly governs the cavitation extension process while the thermal effects are not intense. The influences of the thermal effect start to become dominant as the temperature increases above approximately 58 °C. The reason for this sharp increase of $\Sigma^{*}$ is the variation of the temperature-dependent physical properties, especially the change of liquid/vapor density ratio $D=\rho_{v}/\rho_{l}$ [45]. The effectiveness of applying Σ in cavitating flows to describe the impact of the thermal effect is supported by the literature [45], [46].

However, the influences of the flow characteristics, such as the pressure difference and viscosity, are not included in the thermodynamic parameter. Therefore, the combined suppression parameter (*CSP*) is provided in Eq. 20 to quantify the “dynamic suppression” subjected by various cavitating liquids in geometrically similar machines.

(20) CSP=tanσ2Re^+Σ∗·Sig(ΔT),where,Sig(ΔT)=C1+e-T-Ttrans

In Eq. 20, the first term represents the suppression effect coming from (1) the pressure term, i.e., the square of σ, which is based on the classical second-order cavity length law [21], and (2) the turbulent term, i.e., the reciprocal of Re^ [43], which is scaled by dividing *Re* by $10^{5}$ to be of the same order as Σ*. In addition, the tangent function is used to amplify the effect of the first term based on experimental observation. The sigmoid function in the second term is used as a switch function to activate or deactivate the thermal effect between inertial mode (*T*< $T_{\mathrm{trans}}$) and thermal mode (*T>*$T_{\mathrm{trans}}$). This *CSP* is used to predict the trend of cavitation length variation along with the temperature. Empirical parameters like $T_{\mathrm{trans}}$ (the transition temperature at peak cavity length), *C* (equal to 0.46 here to adjust the scale between the second and first terms) shall be determined by experimental results as detailed in Section 4.2.

## 3 Experimental setup and methods

**Fig. 3:**
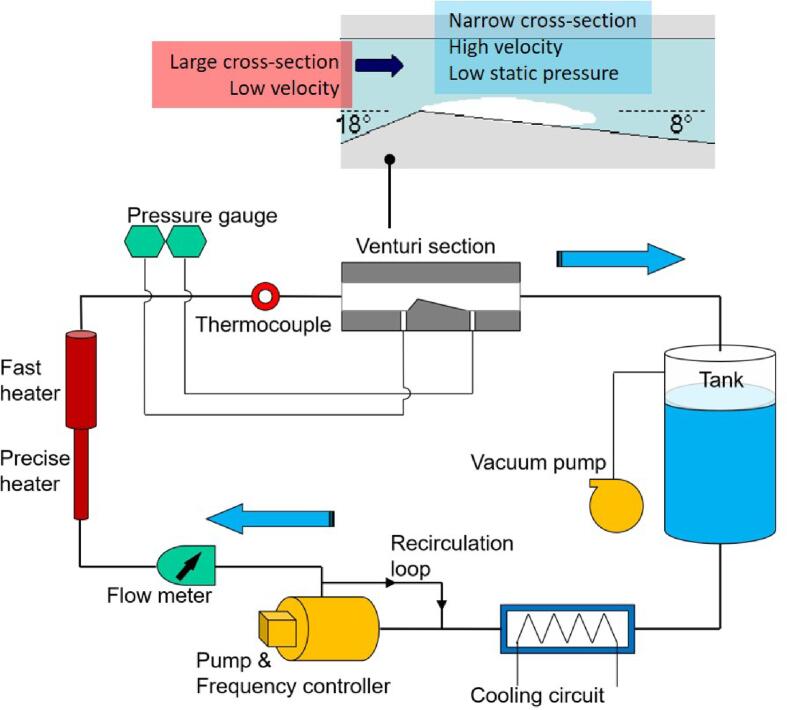
Schematic of hydraulic water tunnel and enlarged drawing of Venturi-type channel.

As shown in Fig. 3, the studied cavitation is generated in the hydraulic loop accompanied by a Venturi tube with the flow direction indicated. The flow rates can be adjusted by the frequency changer connected to a circulating pump (Salmson-Multi-He1602, maximum flow rate 500 L/min, maximum delivery pressure 10 bars). To avoid the pump operating in an unstable condition, a small flow rate can be maintained by the secondary re-circulation loop. The pressure inside the entire loop can be raised with compressed air or decreased by a vacuum pump. A cooling loop together with two heating devices (TEMPCO CHF-02100, 3000 W) are used to adjust the flow temperature. The OMEGA-CLAD type-K thermocouple (temperature range from 0 to 180 ± 0.5 °C), locating close enough to the upstream of the test section, is used to measure the freestream temperature. All the pipes are thermally protected with vacuum insulation. To diminish the effects of non-condensable gas and ensure saturated liquid, a perlator aerator is installed at the water hose with circulating freshwater.

The enlarged Venturi profile shows the hydraulic flow channel with a 5-mm width and 21-mm height $H_{\mathrm{ve}}$ at the channel inlet. With the 18°convergent inlet and 8°divergent outlet, a 10-mm ($H_{\mathrm{th}}$) height throat is formed. The pneumatic tubes linking the pressure gauges (0–20 bars with 0.25% uncertainty, Rosemount 3051T) are connected to the sidewalls locating 50 mm upstream and 90 mm downstream until the throat is reached. The details of test sections can be referred from previous works [47].

To illustrate the thermal effects in a wide temperature range, cases are obtained at water temperatures of 28 °C, 35 °C, 45 °C, 50 °C, 52 °C, 54 °C, 56 °C, 58 °C, 60 °C, 64 °C, 68 °C, and 72 °C. Under five distinctive flow rates, i.e., 36 L/min, 39 L/min, 42 L/min, 45 L/min, and 48 L/min, the experimental setting specifications are shown in Table 1. Note that the temperature interval is slightly larger in Set 5 due to measurement limitations. The cavitation number remains the same in each of the five experimental sets by adjusting the reference pressure. The reference pressure was obtained from the pressure gauge connected to the sidewall locating 50 mm upstream from the throat. The Reynolds number is calculated as Re=ρT∞UrefHthμT∞, where $H_{\mathrm{th}}$ is the characteristic length, which is 10 mm here, the same as the channel height at the throat. In addition, $\mu \left(T_{\infty }\right)$ is the dynamic viscosity at the flow temperature. Assuming that the temperature error affects the water properties of viscosity, density, and vapor pressure in the simplified first-order linear relation, the derivative uncertainty for cavitation number is 3.3% and for Reynolds number 2.2%.

**Table 1:** Flow conditions for five experimental sets.

Set No.	Cavitation No. σ	Flow rate (L/min)	Velocity (m/s)	Upstream pressure (bars)	Reynolds No. ($\times 10^{5}$)	Temperature (°C)
1	1.49	36	12	1.09∼1.39	1.44∼2.99	28∼72
2	1.40	39	13	1.21∼1.50	1.56∼3.24	28∼72
3	1.37	42	14	1.37∼1.65	1.68∼3.49	28∼72
4	1.34	45	15	1.55∼1.83	1.80∼3.74	28∼72
5	1.31	48	16	1.71∼1.98	1.92∼3.99	28∼72

A Photron Fastcam SA1.1 camera (with 100-mm TokinaF2.8D lens mounted) was used for high-speed visualization. The maximum recording speed is up to 675,000 fps (frames per second) at reduced resolutions, or 5,400 fps at full resolution (1024p×1024p). In Table 2, three imaging configurations are listed, i.e., (1) side view with top-light illumination, (2) side view with backlight illumination, and (3) top view with backlight illumination of the test section. The acquisition rate of top-light illumination is 8,000 fps, which can be extended to 20,000 fps for backlight illumination without causing severe overexposure. The exposure times were 59 and 1.7 μs for top-light and backlight illuminations, respectively.

**Table 2:** Visualization parameters under three illumination techniques.

Camera setting	Side-view top light	Side-view backlight	Top view
Resolution	900×240 pixels	900×240 pixels	1024×288 pixels
Acquisition rate	8,000 fps	20,000 fps	20,000 fps
Shutter speed	1/17,000 s	1/593,000 s	1/593,000 s
Pixel size	81μm×81μm	81μm×81μm	53μm×53μm
Active area	72.9 mm×19.4 mm	72.9 mm×19.4 mm	54.3 mm×15.3 mm

## 4 Results and discussion

### 4.1 Unsteady cloud-shedding behavior of cavitation

**Fig. 4:**
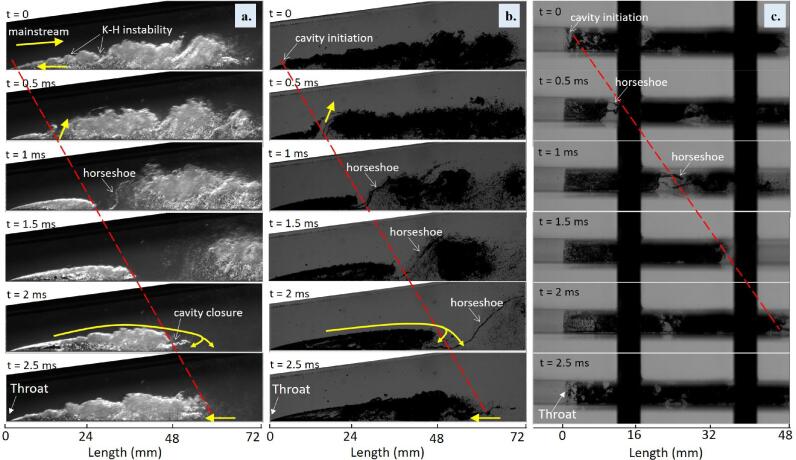
Temporal evolution of cloud cavitation case σ = 1.15 at T = 65 °C with (a) side view top lighting; (b) side view backlighting; (c) top view backlighting.

Fig. 4 shows the classical dynamic behavior of the cavity, known as “cloud-shedding cavitation,” from three imaging settings. Under top lighting in Fig. 4(a), the high void fraction areas (vapor phase) generate a higher gray level as the light beam is refracted at the liquid–vapor interfaces. However, in Fig. 4(b) with backlighting, white regions represent the liquid phase and black regions are the cavity area, since the vapor phase could block the light beam. It is shown that the backlighting image shows a distinct exterior shape of the cavitating zone. Conversely, top lighting provides details of the interior vapor structures due to its larger gray-level gradients.

Overall, the characteristic stages of cavitation growth are (1) the attached cavity sheet develops from the Venturi’s throat; (2) as the attached cavity is growing, a re-entrant flow is generated; (3) the sheet cavity is disturbed and small bubble clusters break up at the closure; (4) the main cloud detaches from the rear part of the cavity, moves downward, and then collapses at the high pressure downstream. Taking Fig. 4(a) as an example, the cavity evolution starts when the re-entrant jet (yellow arrows) reaches the throat area at t = 0. The re-entrant jet is an upstream-spreading, thin viscous film moving over the bottom wall reverse to the sheet movement [48]. It is initiated as a thick mixture flow at the cavity closure and can be observed as a dark gray level (shown by the arrow at t = 2.5 ms). When the jet film arrives at the upstream throat, it becomes too thin to be observed, but the upstream re-entrant jet, together with the downstream main flow, makes the vapor–liquid interface wavy or vortices-shaped, due to the Kelvin–Helmholtz (KH) instability, shown at t = 0.

The thickness of the cavity at this throat area is sufficiently thin that the re-entrant could cut off the interface (indicated by arrows at a time of 0.5 ms). The cavity of maximum length is assumed to be just before this cutoff, since the cutoff means the detachment of the prior cavity and the inception of the new cavity. It even looks like the cavity still grows as one integral piece, and a hollow horseshoe structure forms (see Fig. 4c) and connects the top of the detached cavity and the bottom of the initiated cavity.

From t = 1 to 2 ms, the horseshoes are stretched into a long, thin chain of bubbles. This is because the growth of the upstream cavity sheet is slower compared with the motion of the detached cavity, which moves following the mainstream flow velocity. At t = 1.5 ms, the bottom of the horseshoe breaks up, and the upper parts merge into the downstream clouds. At this stage, the cavity sheet grows with a well-defined outline and attaches to the bottom wall, while the detached vapor clusters move downstream, forming a round shedding cloud. Regarding the outside mainstream fluid, it is choked away from the bottom wall by the vapor and then re-attaches to the surface when approaching the cavity closure area, as shown by the yellow arrows at t = 2 ms. As modeled by [49], the mainstream flow will impinge onto the bottom wall and separate. A small portion that will become the re-entrant jet travels upstream due to the reverse pressure gradient, and the other continues moving downstream. Here, the cavity sheet grows steadily until the re-entrant jet forms, leading to the cavity detaching from the bottom wall at the closure area shown by arrows at t = 2.5 ms. In the next 0.5 ms, the reentrant jet moves upstream, reaching the throat area, and a new cavity evolution cycle restarts approximately at time t = 3 ms.

### 4.2 Effect of temperature on change of cavity length

**Fig. 5:**
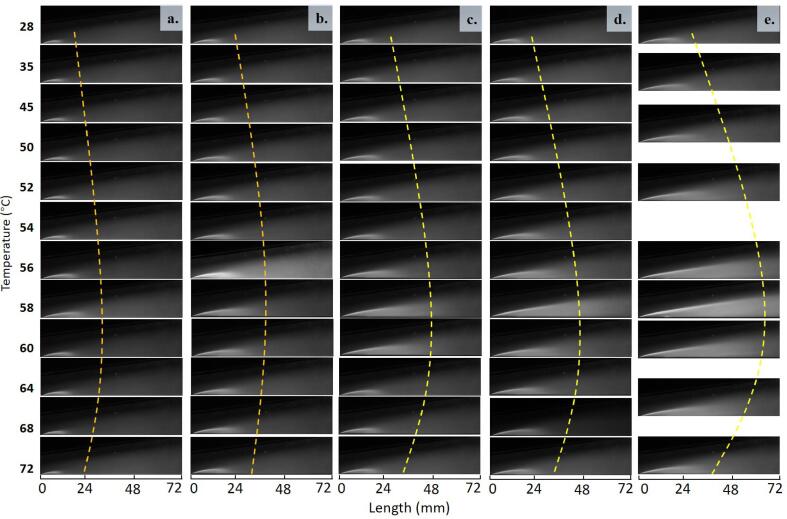
Time-averaged cavitation plots for five cavitation numbers: (a) σ = 1.49; (b) σ = 1.40; (c) σ = 1.37; (d) σ = 1.34; (e) σ = 1.31.

To evaluate the effect of temperature on the extent of cavitation, the time-averaged gray level images under top lighting of all the cases are shown in Fig. 5. All the captured images were first normalized by the reference image $I_{\mathrm{ref}}$ taken at non-cavitation conditions. They are then converted from 16-bit into 8-bit gray images for the convenience of analysis, with gray levels ranging from 0 (black) to 255 (white). Finally, the mean value of gray level, $\overset{‾}{I}$, is calculated by $\overset{‾}{I}=\frac{1}{N}\sum_{i=1}^{N}\left(I_{i}-I_{\mathrm{ref}}\right)$, where N = 2,000 images, which consist of over 100 shedding cycles.

As can be seen in Fig. 5, the time-averaged cavitation plots show the general shape of cavitation with dynamic information eliminated. From the left to the right column, the cavitation number σ decreases from 1.49 to 1.31, with cavitation length increasing accordingly. From the top to the bottom row, the temperature increases from 28 °C to 72 °C to confirm the cavity-size-changing trend. In Set 5 (σ = 1.31), measurements were taken with a larger temperature interval due to experimental limitations. With increasing temperature, the cavitation length first increases and then decreases to very short, shown by the yellow dashed trend lines. The cavitation length peaks of different flow rate cases appear at approximately 58 °C. The so-called thermal transition in thermo-fluids can be observed from this transition process as the temperature increases [50]. Such a convex trend of the ”first increasing then decreasing” phenomenon has also been reported in researching cavitation erosions [27], [28].

**Fig. 6:**
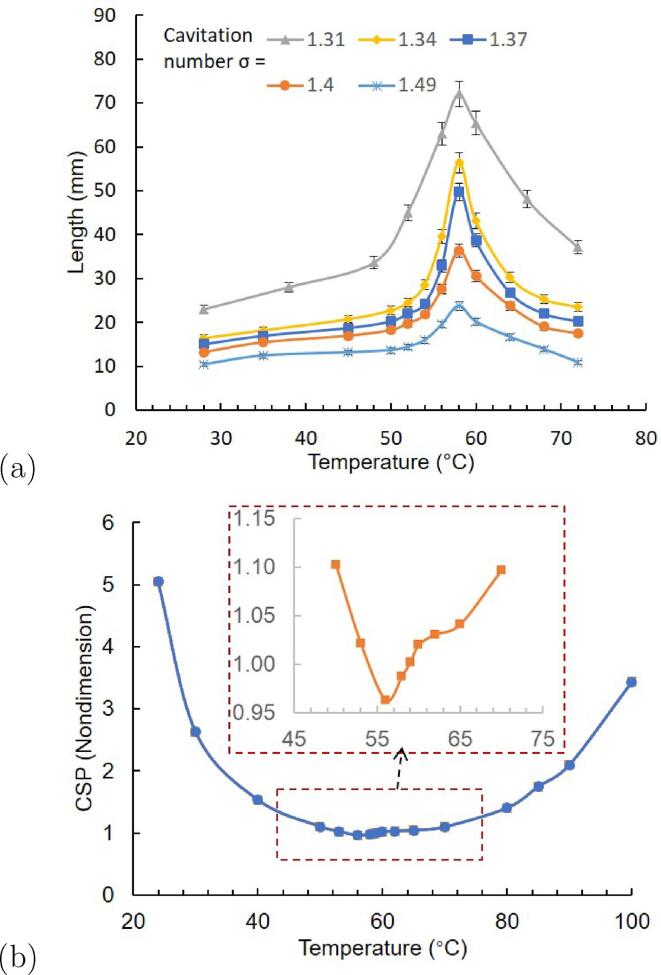
Correlation between cavity length and CSP value along with temperature. (a) Cavitation length growth trend at five cavitation numbers; (b) variation of CSP value with enlarged view at temperature range of 45 °C to 70 °C.

Fig. 6a presents the variation of cavity length $L_{\mathrm{cav}}$ along with temperature change at five cavitation numbers. The cavity length is determined at the closure of the time-averaged cavity outline, which is extracted based on the “maximum standard deviation threshold” as detailed in [51]. The uncertainty on $L_{\mathrm{cav}}$ is inferior to 2%, according to the convergence analysis performed with various parameters, such as the diverse number of images used, effects of the gray level thresholds for the binarization and filters used for snapshots, etc. Results also show that the maximum cavitation length, for five cavitation numbers, appears at approximately 58 °C, which is determined as the transition temperature $T_{\mathrm{trans}}$. As indicated by Chen [50], the thermo-sensitive mode (beyond $T_{\mathrm{trans}}$) and the inertial mode (below $T_{\mathrm{trans}}$) can be distinguished depending on whether thermal effects play a dominant role or not.

Inserting $T_{\mathrm{trans}}$ = 58 °C into Eq. 20, the effect of the CSP can be seen in Fig. 6b. The values of parameters used to calculate the CSP at 58 °C is listed here as an example, i.e., the density of the vapor $\rho_{v}$ = 0.1188 $\mathrm{kg}/m^{3}$, density of the liquid $\rho_{l}$ = 984.2 $\mathrm{kg}/m^{3}$, latent heat of vaporization *L* = 2.36e6 J/kg, liquid specific heat $c_{\mathrm{pl}}$ = 4184 J/(kg·K), Kelvin temperature $T_{\infty }$ = 331.15 *K*, and thermal diffusivity of the liquid $\alpha_{l}$ =1.63e-7 $m^{2}/s$. The values of water/vapor properties are referred to the NIST-REFPROP database.

At lower temperatures (T < 56 °C), the combined suppression effect is lessened (due to σ↓ or *Re*↑) as the temperature increases. The cavitation growth is mainly driven by the vaporizing processes, in addition to the fact that the thermal effects are not very remarkable. The intensified vaporization is due to (1) the smaller pressure difference (σ↓) between the ambient pressure and the vapor pressure, and (2) the turbulent mixture effect (*Re*↑) leading to the larger bubble interface area. Thus, the large volume of water at the water–vapor boundary is vaporized, supporting further cavitation growth. This latent heat must be supplied from the cavity surroundings, and the resulting requirements for larger heat transfer decrease the local water temperature. At higher temperature (over 56 °C), the influence of thermal effect surge (Σ↑) becomes dominant and decisive compared with the vaporization process. The temperature decreases of water result in local decreases of vapor pressure, which suppress cavitation again [44]. As a result, the growth of the bubble is delayed and weakened. The thermal delay phenomenon is predicted to be dominant at 56 °C, which is slightly unusual from the experimental result at 58 °C. Admittedly, the *CSP* parameter, including the constant C, is determined based on the limited experimental data and needs further validation. Hence, the provided estimation of the cavity length trends may be specific to the cavitating flow in the currently investigated small Venturi channel.

### 4.3 Effect of temperature on shedding regimes of cavitation

**Fig. 7:**
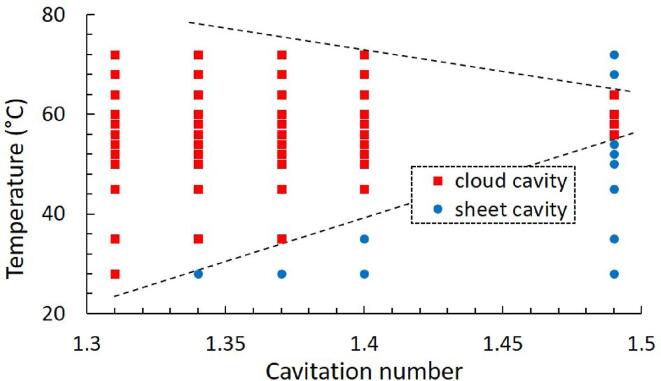
Classification of cavitation shedding regimes: cloud-cavity type (red rectangular dots) and the attached-cavity type (blue round dots).

Depending on the dynamic behavior of cavitation, all the cases can be classified into two regimes: the attached sheet-cavity type (round dots) and the detached cloud-cavity regime (rectangular dots), as shown in Fig. 7. Roughly speaking, the detached cloud cavities appear on the left-hand side of two dashed lines as illustrated. As discussed in other studies [21], [48] and observed here, at sufficiently low cavitation number or high temperature the shedding process transitions from sheet cavity to cloud cavitation due to the changing of cavity length. With the length extension, the cavitation regime transitions from sheet cavity to cloud cavity (see cases of σ = 1.49 at 28 °C and 58 °C). Then, over the transition temperature, the cavity types tend to transform back when suppression effects occur. In the sheet-cavity regime, the upstream cavity sheet remains attached to the wall while only the closure length undergoes oscillations. The sheet cavitation can be considered to be quasi-steady, compared with cloud cavitation, in which the entire cavity sheet is detached from the wall and shed as clouds.

**Fig. 8:**
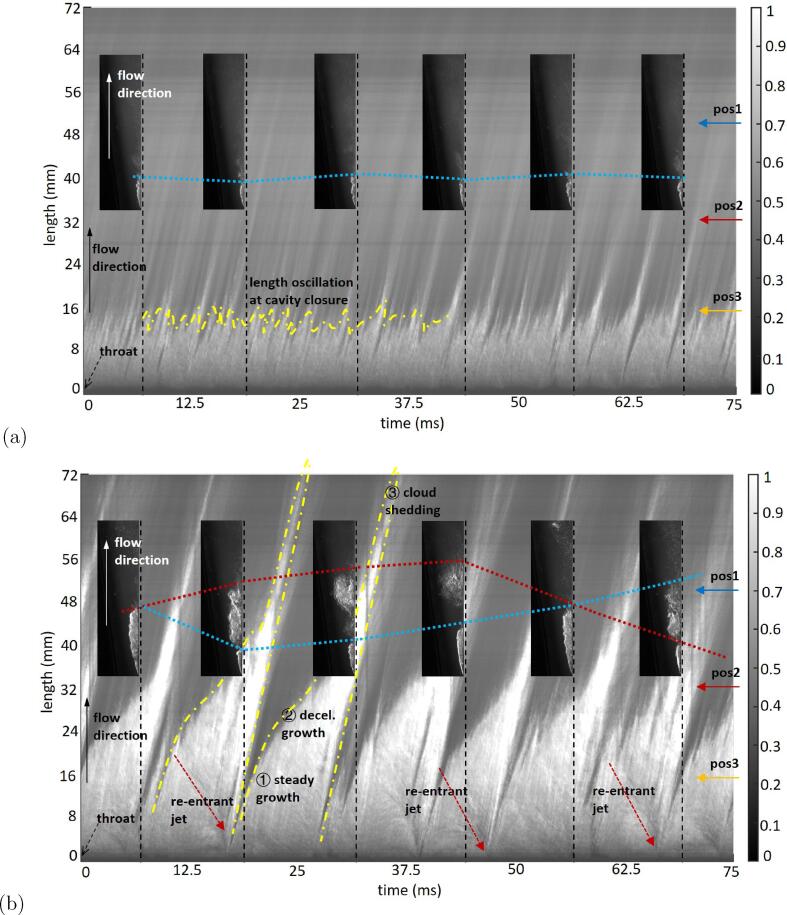
Spatio-temporal distribution diagram derived from 1,000 consecutive cavitation snapshots for cases σ = 1.15 at (a) 28 °C and (b) 58 °C.

To highlight the dissimilarity among different shedding regimes, the spatio-temporal evolution of cavitation is demonstrated by “squeezing” a two-dimensional frame into a one-dimensional column. For each frame, the gray level at the cavitation area can be averaged along the cross-section to keep the dimension of the flowing direction alone. Combining the frames of the entire-time range and presenting them in an abscissa-time, ordinate-position graph, the light transmission changing from prior snapshots to the next is illustrated (Fig. 8). In this work, the temporal development of case σ = 1.4 at temperatures of 28 °C and 58 °C within 75 ms (1,000 frames with a time interval δt=125μs) are shown as an example.

The cavity starts from the Venturi’s throat at a length of 0 mm. With top lighting, the cavitation area refracts light and is brighter (normalized gray level tends to be 1), compared with the dark area of the liquid phase (normalized gray level tends to be 0). In Fig. 8(a), the main part of the cavity sheet is continuously attaching to the bottom wall. Small horseshoe clusters are shed out and collapse immediately at the cavity tail, leading to the trailing fluctuations indicated by the yellow line. Therefore, the sheet-cavity length is quasi-steady, except at the closure area.

By comparison, the intuitive characteristics of cloud cavitation are the larger cavity size (cavitation length over 30 mm) and the clear periodicity (approximately eight shedding processes during 75 ms). The yellow dashed line indicates one single shedding process of cloud cavitation that contains three stages: (1) steady growth, during which the cavity grows linearly along time starting from the inception occurring near the Venturi’s throat; (2) decelerated growth, i.e., the cavitation length grows more slowly since the disturbance of the emerging re-entrant jet; and (3) cloud shedding in which the detached clouds are accumulated and rolled towards the downstream. The delimitation between steady growth and decelerated growth is the initiation of a re-entrant jet, the movement of which is indicated by the red dots and arrow. When this re-entrant jet moves close to a throat, the tail part of the cavitation tends to detach from the bottom wall and shifts into a detached cloud in the downstream direction. The cavitation frontal sheet part grows and shrinks periodically, while the posterior cloud part moves downstream and collapses, as indicated by the blue and red dashed lines in Fig. 8(b).

**Fig. 9:**
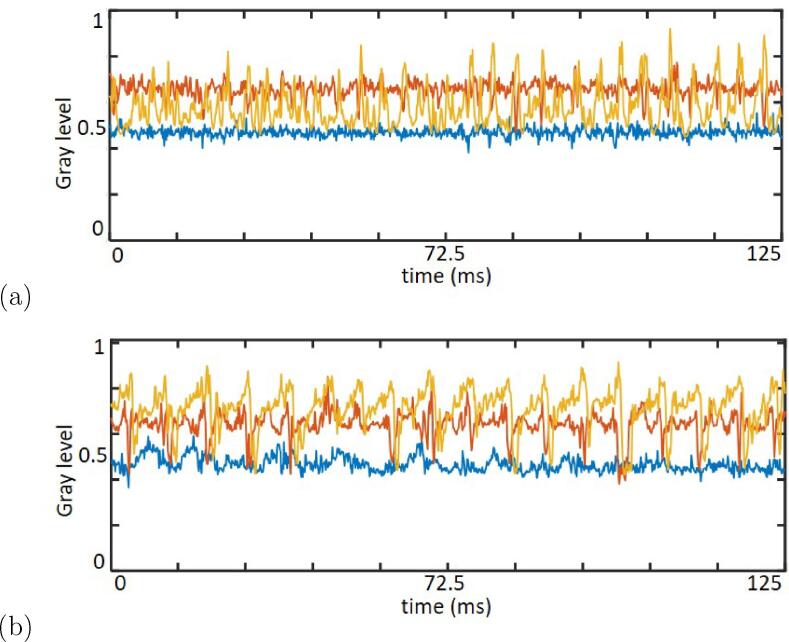
Time evolution of gray level fluctuation at three positions (positions 1, 2, and 3 at lengths 48, 32, and 16 mm, respectively, indicated in Fig. 8) for cases σ = 1.4 at (a) 28 °C and. (b) 58 °C.

To study the periodicity of cavitation shedding behavior, the fluctuations of gray level during 125 ms are extracted as shown in Fig. 9. The gray level fluctuations at three positions (positions 1, 2, and 3 at lengths 48, 32, and 16 mm, respectively) are averaged within a 2-mm width and plotted along the time. Position 3 is near the closure area of the sheet-cavity case, while position 1 is in the downstream zone over the cavity zone. The unsteady cavitation characteristics can be analyzed from their oscillation frequencies.

**Fig. 10:**
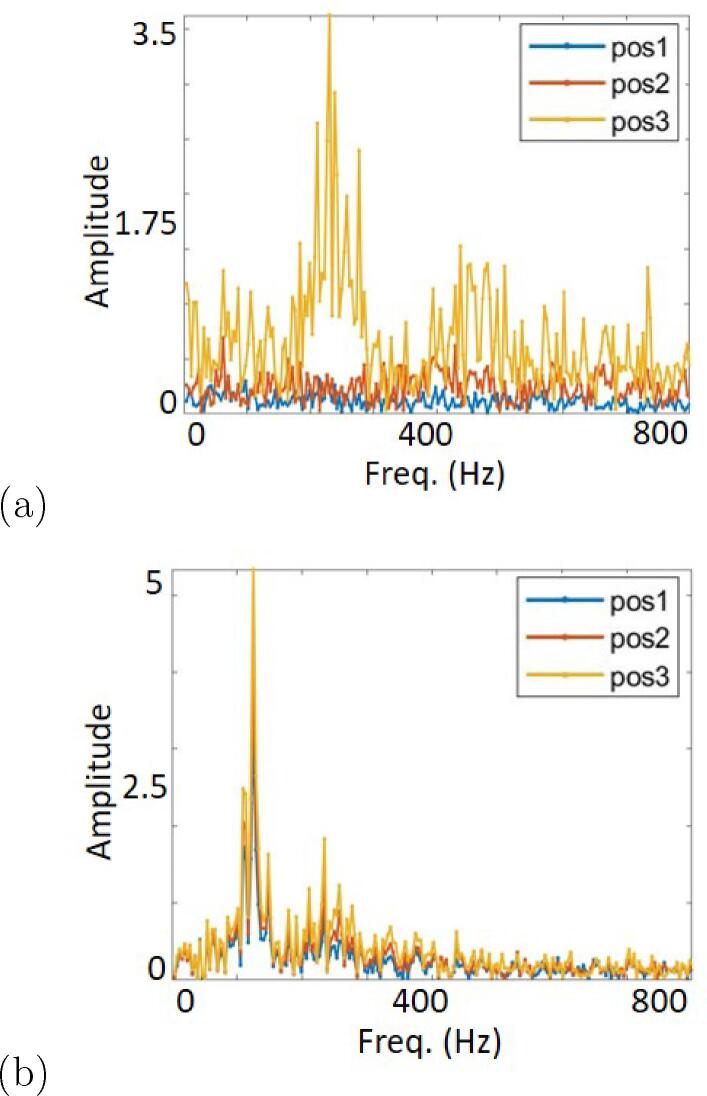
Cavitation shedding spectra of cases σ = 1.4 for (a) sheet cavitation at 28 °C and (b) cloud cavitation at 58 °C. Ordinate is the spectra amplitude and abscissa the frequency (Hz).

It can be seen from the above results that cavitation oscillates in certain periodic behavior. A fast Fourier transform (FFT) is performed on gray level fluctuations to calculate the characteristic shedding frequencies *f*. In Fig. 10(a), a primary frequency appears at position 3, while no characteristic frequency is detected at positions 1 or 2 due to the lack of cavity structures in these areas of the sheet-cavity case. With increasing temperature, the sheet cavity transformed into a cloud cavity. As the cavitation length increases, the primary frequency is reduced from 230.5 Hz at 28 °C to 106.7 Hz at 58 °C. In addition, contrary to Fig. 10(a), the characteristic frequency of shedding exists at all three positions of the cloud-cavity case in Fig. 10(b). Moreover, the amplitude of f is larger in the cloud-cavity case than in the sheet-cavity case. This implies that upon comparison with cloud cavitation, which consists of apparent temporal coherent structures, the small vapor shedding at the sheet cavitation closure occurs more irregularly.

## 5 Conclusions

In this study, the combined suppression effects of cavitation number (pressure difference), Reynolds number (turbulent level), and thermodynamic parameter (temperature) on the intensity and spatio-temporal characteristics of hydrodynamic cavitation were investigated. Through theoretical analysis and experimental validations, the variation trends of cavitation length were quantitatively identified at different temperatures, as well as cavitation evolution cycles, shedding regimes, and vapor ratio fluctuating properties affected by suppression mechanisms. With increasing temperature, the averaged cavity length increased until $T_{\mathrm{trans}}$ = 58 °C and then decreased. It has been widely considered that the significant thermodynamic effects lead to a shorter, thinner attached cavity in higher-temperature water. However, the magnitude of thermal suppression was impacted not only by the thermal parameter Σ(T) but also by the Reynolds number and cavitation number. The *CSP* value was provided to predict the cavity length peak at approximately $T_{\mathrm{trans}}$.

Owing to the link between the cavity length and its dynamics, the cavitation first increased and tended to transition from sheet cavitation to cloud cavitation with increasing fluid temperature. However, at an excessive temperature increase (over $T_{\mathrm{trans}}$), the cavity length decreased and transitioned back to sheet cavitation. Therefore, the type of cloud cavity could be maintained by adjusting the flow temperature, as it had a higher aggressiveness and is desired in utilizing the cavitation treatment effects. The cavity length also affected the characteristic frequency of the quasi-cycles of shedding, i.e., a higher frequency was obtained from the smaller-size cavity. As the cavitation size was proved to be the dominant parameter, the shedding frequency showed a converse trend of “decreasing and then increasing” as the temperature increased.

## CRediT authorship contribution statement

**Mingming Ge:** Conceptualization, Data curation, Formal analysis, Writing - original draft. **Chuanyu Sun:** Investigation, Supervision, Methodology, Writing - review & editing. **Guangjian Zhang:** Software, Validation, Visualization, Writing - review & editing. **Olivier Coutier-Delgosha:** Funding acquisition, Project administration, Resources. **Dixia Fan:** Validation, Writing - review & editing, Funding acquisition.

## Declaration of Competing Interest

The authors declare that they have no known competing financial interests or personal relationships that could have appeared to influence the work reported in this paper.
